# Role of Intranasal Steroid in the Prevention of Recurrent Nasal Symptoms after Adenoidectomy

**DOI:** 10.1155/2013/603493

**Published:** 2013-08-04

**Authors:** Tamer S. Sobhy

**Affiliations:** Faculty of Medicine, Ain Shams University, 15 Khalifa Maamoon, Heliopolis, Cairo, Egypt

## Abstract

*Background*. Intranasal steroid provides an efficient nonsurgical alternative to adenoidectomy for theimprovement of adenoid nasal obstruction. *Objective*. To demonstrate the role of intranasal steroid in the prevention of adenoid regrowth after adenoidectomy. *Methods*. Prospective randomized controlled study. Two hundred children after adenoidectomy were divided into 2 groups. Group I received postoperative intranasal steroid and group II received postoperative intranasal saline spray. Both medications were administered for 12 weeks postoperatively. Patients were followed up for 1 year. Followup was done using the nasopharyngeal lateral X-rays, reporting the degree of the symptoms. *Results*. Significant difference between both groups after 6 months and after 1 year. The intranasal steroid group had significantly lower score after 6 months and after 1 year as regards nasal obstruction, nasal discharge, and snoring than the intranasal saline group. 2 weeks postoperatively, there was no difference between both groups as regards nasal obstruction, discharge, or snoring. As regards lateral radiographs, there was statistically significant difference between both groups 1 year but not 6 months postoperatively. *Conclusion*. Factors influencing the outcome of intranasal steroids therapy in the prevention of adenoid regrowth have not been identified. However, this treatment may obtain successful results in children to avoid readenoidectomy.

## 1. Introduction

Nasal obstruction is one of the main symptoms of adenoid hypertrophy; they are also presented with chronic rhinorrhea, snoring, hyponasal speech, and obstructive sleep disorder [1]. Adenoidectomy can reduce both nasal obstructions and upper respiratory infections. However, some patients display clinically significantly persistent nasal symptoms even after surgery. Symptoms, such as nasal obstruction or recurrent upper respiratory infections, persist in 19–26% of patients [2]. Adenoidectomy remains a commonly performed procedure, although it produces short-term benefits [3]. There are 2 difficulties that have been described to prevent complete adenoidal removal. Firstly, lymphoid tissue in the pharyngeal recess is considered by all authors as difficult to remove [4]. The second difficulty is the bulging adenoidal tissue into the posterior choanae, which was addressed by Pearl and Manoukian [5]; they found choanal adenoids in 9% of their study group.

Although there are few nonsurgical alternative treatment options, these may be considered in less serious cases. Accordingly, studies about intranasal steroid applications under various protocols have been presented in the literature, but none of these studies addressed the efficacy of intranasal steroids to prevent recurrence of adenoid after adenoidectomy.

## 2. Patients and Methods

This study was a prospective randomized controlled parallel clinical study. As the study had no connection with any of the manufacturers of the drugs or the pharmaceutical industry at all, it was not possible to obtain a placebo, and therefore the study could not be double blinded. Simple randomization was done with every other patient consecutively.

The study was approved by the Institutional Review Board of the Otorhinolaryngology Department, Faculty of Medicine, Ain Shams University, Cairo, Egypt. Children presented to ENT outpatient clinic at Ain Shams University hospitals during the period from April 2009 to June 2011, diagnosed as adenoid hypertrophy, were included in the study. The study included 2 groups; each included 100 children. Written informed consents from the parents were taken about the participation of their children in the study.

The diagnosis was based on the following symptoms: nasal obstruction, nasal discharge, and/or snoring and lateral radiographs (enlarged convex bulge in the roof of the nasopharynx compressing the nasopharyngeal airway). Exclusion criteria included the use of intranasal or systemic steroids within the last 1 year, use of any intranasal medication within the previous 2 weeks of entering the study, acute URTI within 2 weeks of entering the study, history of epistaxis, immunodeficiency disorders, or hypersensitivity to the mometasone furoate. Also, children were excluded from the study if there is a history of craniofacial neuromuscular or genetic disorder.

Assessment of each child upon entering the study included the following: history and physical examination, parental questionnaire, and lateral nasopharyngeal radiograph. All patients under the study had complete head and neck evaluation, including flexible fiber-optic nasal endoscopy (according to the compliance of the child). Due to the difficulty of the use of the flexible endoscopy for all patients, it was not feasible to be used in the assessment of the nasopharynx pre or postoperatively.

The patients were all assessed before and after adenoidectomy as regards the nasal obstruction, discharge, and snoring. These symptoms were all graded as Grade 1: mild, Grade 2: moderate, and Grade 3: severe.

The lateral view nasopharyngeal radiograph, the size of the adenoids was graded according to the palatal airway measured from the most convex point of the adenoid tissue to the soft palate. The narrowest distance between the nasopharyngeal soft tissue and the soft palate was taken. Grading was as follows, Grade 1: >6 mm; Grade 2: 4–6 mm; Grade 3: 0–3 mm. The lateral radiographs were done before surgery and 6 months and 12 months postoperatively. 

The parents were asked (using the questionnaire) about their overall satisfaction of the nasal condition of the child after adenoidectomy. They were also asked about the number of upper respiratory tract infections in the previous years and postoperatively.

 Adenoidectomy was done using the classical method using the adenoid curette. After adenoidectomy, children were then simply randomized into 2 groups, group I which included (100 children) with postoperative intranasal steroids and group II (100 children) who had postoperative intranasal saline spray. Patients in group I received 12-week course of single intranasal spray administration in each nostril with mome tasone furoate (40 mcg/day). After this course, all patients in group I were reassessed to evaluate the efficacy of treatment. All patients or parents were asked to report the degree of the symptom after 2 weeks, 6 months, and 1 year postoperatively with the questionnaire that is fulfilled by the parents. No other medication was allowed during the treatment. Patients who used systemic steroids for any other reason were excluded from the study. Patients in group II received intranasal saline nasal spray for the same period (12 weeks), and assessment was done in the same way as group I. All patients were followed up after adenoidectomy after 2 weeks, 6 months, and 1 year. Lateral radiographs were done after 6 months and after 1 year in the postoperative period.

*Statistical Analysis.* The collected data was revised, coded, tabulated, and introduced to a PC using statistical package for Social Science (SPSS 15.0.1 for windows; SPSS Inc., Chicago, IL, USA, 2001).

## 3. Results

In group I patients with intranasal steroids, there were 96 patients (4 patients were excluded from the study) with age range from 3 to 13 years (mean age = 7.42 years), and there were 54 males (56.3%) and 42 females (43.8%), while in group II with intranasal saline included 92 patients (8 patients were excluded) with age range from 3 to 13 years (mean age = 5.89 years); this group included 42 males (45.7%) and 50 females (54.3%). Demographic data of the patients in both groups are shown in Table 1.

As regard nasal obstruction, highly significant difference towards group I with intranasal steroids, when compared with group II with intranasal saline 6 months (*P* = .001) and 1 year (*P* = .031) postoperatively. However, 2 weeks in the postoperatively period, there was no significant difference between both groups.

Regarding nasal discharge, there was a highly significant difference (*P* = .0001) between both groups, towards group I with intranasal steroids after 6 months and after 1 year (*P* = .001), in the postoperative period. While after 2 weeks in the postoperative period, there was no significant difference (*P* = 1.00) between both groups.

Regarding snoring, there was a highly significant difference between both groups after 6 months (*P* = .0001) and after 1 year (*P* = .001). There was no significant difference after 2 weeks (*P* = .363) in the postoperative period. 

**Figure 1:**
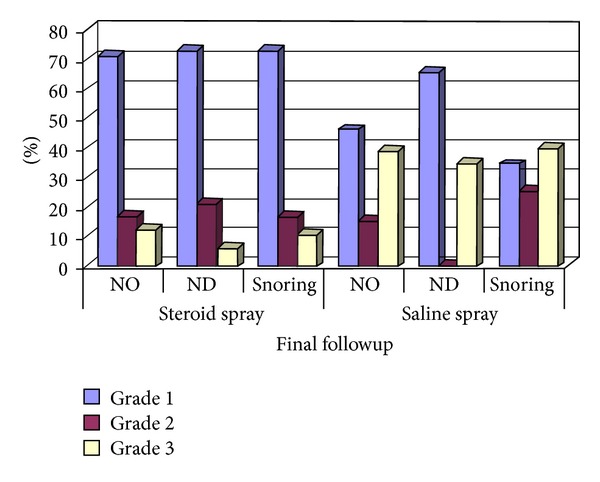
Description of nasal symptoms grades among steroid and saline patients at the last followup, 1 year postoperatively. This graph shows the differences between the nasal symptoms grades between both study groups after 1 year (NO: nasal obstruction, ND: nasal discharge, Grade 1 = mild, Grade 2 = moderate, and Grade 3 = severe).

In group I, comparing data after 1 year with the data of the preoperative period, there was a highly significant difference elicited in all the 3 symptoms (*P* = .0001). Comparing data after 1 year with the data after 2 weeks in the postoperative period, there was significant difference regarding nasal obstruction only (*P* = .0001) but no difference regarding nasal discharge and snoring. When comparing data after 1 year with the data after 6 months, there was significant difference as regards the 3 symptoms with *P* = .011 in nasal obstruction, *P* = .008 in nasal discharge, and *P* = .003 in snoring. This can be illustrated in Figure 1.

Significant difference (*P* = .003) was noted towards group I patients regarding nasopharyngeal radiograph after 1 year in the postoperative period. However, there was no significant difference between both groups (*P* = .191) 6 months postoperatively.

**Figure 2:**
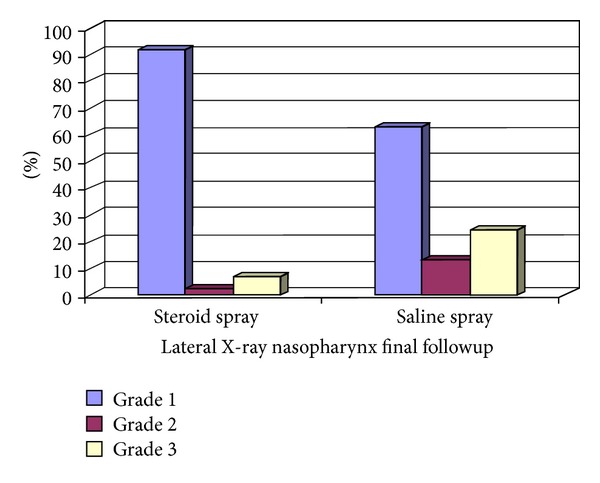
Description of X-ray findings among steroid and saline patients at the last followup, 1 year postoperatively. This graph shows the differences between patients as regards the adenoid enlargement grade between both study groups after 1 year (Grade 1 = >6 mm, Grade 2 = 4–6 mm, and Grade 3 = 0–3 mm).

In group I patients, there was a highly significant difference (*P* = .0001) of the nasopharyngeal radiographs when comparing the data after 6 months with the data in the pre-operative period. A highly significant difference (*P* = .0001) could be elucidated when comparing the data after 1 year with the data in the preoperative period despite failing to find any difference of the radiograph data after 1 year when compared with the data after 6 months which is shown in Figure 2.

In the current study, it was found that the overall satisfaction among parents of group I patients was 85.4%. Overall satisfaction among parents of group II was 76.1%. When comparing the overall satisfaction between both groups, there was no statistically significant difference. Also, there was a highly significant difference of the number of URTI when comparing data of the pre- and postoperative periods. The same data was found in group II with a significant difference (*P* = .0001).

There were no intraoperative or postoperative complications in group I. While in group II, post operatively, 3 patients developed secondary bleeding after 7 days.

## 4. Discussion

Revision adenoidectomies are not unheard of. However, a review of the literature, including some prominent textbooks, does not illuminate the issue or its frequency [4]. Buchinsky and coworkers [4] failed to find a new obstructing adenoid pad after adenoidectomy in a large series of children, while, on the contrary, Joshua and his colleagues in [2] found a new obstructing adenoid tissue in the clinical practice. They reported infrequent occurrence of adenoid re-growth after adenoidectomy that causes nasal obstruction which accounts for 3% of patients with persistent postadenoidectomy symptoms.

Successful use of intranasal steroid treatment in children with adenoid hypertrophy was introduced by Demain and Goetz [6]. Although it is not yet clear by which mechanisms the steroids reduce the nasal airway obstruction, however, there are some theories such as the anti-inflammatory effect of steroids that help to reduce adenoidal and nasopharyngeal inflammation [6].

The present study showed that the use of intra nasal steroids after adenoidectomy was beneficial to relieve nasal obstruction and prevent recurrence of adenoid after adenoidectomy after a follow-up period of 1 year. There is a difference in age across groups (7.4 in group I and 5.89 in group II) which might account for the difference across groups as the Waldeyer's ring involutes with puberty. However, Buchinsky and colleagues [4] failed to find any significant difference between children younger than 10 years and those older than 10 years as the proportions between both groups were identical.

In the current study we could not perform endoscopy for all children, although it is now the best diagnostic technique for diagnosis of adenoid-related nasal obstruction because it depends on the age and compliance of the child.

The use of postoperative intranasal steroid gives advantage to avoid the 2nd intervention. This is in contrast to Lepcha and coworkers [7], who did not find any significant efficacy of intranasal steroids in improving nasal blockage, nasal discharge, or snoring, although a fivefold reduction in adenoid size was observed in intranasal steroid group when compared with the placebo group. However, this difference did not reach a statistical significance.

Steroids are generally well tolerated in children. Studies showed only one case of episodic nasal bleeding [8]. The effect of intranasal steroids on growth was studied by Allen and his colleagues in [9], in a randomized, double-blind, placebo-controlled study. The growth rate in pre-puberty children who had used intranasal steroids for 1 year was reported to be equal to the growth rate of the placebo control group.

The mechanisms by which topical steroids improve nasal airway obstructive symptoms remain unclear. Three main trials succeeded to demonstrate the improvement of nasal obstruction with reduction of adenoid size with the use of intranasal steroids [6, 10, 11].

Nonsurgical alternatives for adenoid hypertrophy are limited to treatment of the coexisting upper airway infections. However, it was reported that treatment with intranasal steroids can decrease the size of adenoid hypertrophy, using beclomethasone [6], fluticasone [12], and mometasone [13]. Among several commercially available steroid nasal sprays, we selected mometasone furoate for this study. This drug had been reported previously not to cause any adverse effects on growth and hypothalamic pituitary adrenal axis. Also, the systemic availability of the drug after topical administration is lower than that of other steroids [14]. 

Ciprandi and coworkers in [15] found that the use of intranasal flunisolide was associated with a significant reduction of adenoid hypertrophy in 72.6 % of the children. On the contrary, isotonic saline solution was associated with a nonsignificant improvement of adenoid hypertrophy as reported in 30.7% of children. A recent study provided evidence that treatment with nasal steroids could represent for some children an effective means of avoiding adenoidectomy [16]. The current study also clarified similar results as Ciprandi and his colleagues [15], as there was significant reduction in the size of the adenoid in lateral radiographs after 1 year with a *P* value = .003.

In this study, it was found that the overall satisfaction among parents of group I patients was 85.4%. Similar results were reported by Lesinskas and Drigotas [1]; they showed that 82.7% of the parents were satisfied with the results of adenoidectomy.

The duration of treatment with intranasal steroids in previous studies varied from 8 to 24 weeks. None of these trials established the optimal duration of treatment in children. The effects are expected after 2 weeks of the initiation of the treatment as described by Criscuoli et al. [16].

The study is limited by the absence of the nasal endoscopic examination which is considered the best diagnostic technique for the diagnosis of the adenoid-related nasal manifestations. We did not include nasal endoscopy because of the noncompliance of the children. One of the limitations is the noncompliance of the children to the intranasal steroids which is observed mainly in the young children. Another limitation of the study is the method of adenoidectomy which is limited to the adenoid curette, although other methods are used, but the most feasible method of adenoidectomy was the adenoid curette. A multicenter study with longer follow-up periods on the study patients should elicit a more informative data. 

## 5. Conclusion

 Factors influencing the outcome of intranasal steroids therapy have not been identified. However, this treatment may obtain successful results in children to avoid surgery for adenoid recurrence. The most appropriate drug, the most efficient dose, and optimal treatment duration need to be investigated and determined.

## Figures and Tables

**Table 1:** Demographic data of the 2 groups of the study.

		Intranasal steroid group		Intranasal saline group	
		*N*	%	*N*	%
Sex	Male	54	56.3%	42	45.7%
	Female	42	43.8%	50	54.3%
Age	Mean ± SD	7.42	2.86	5.89	2.72
